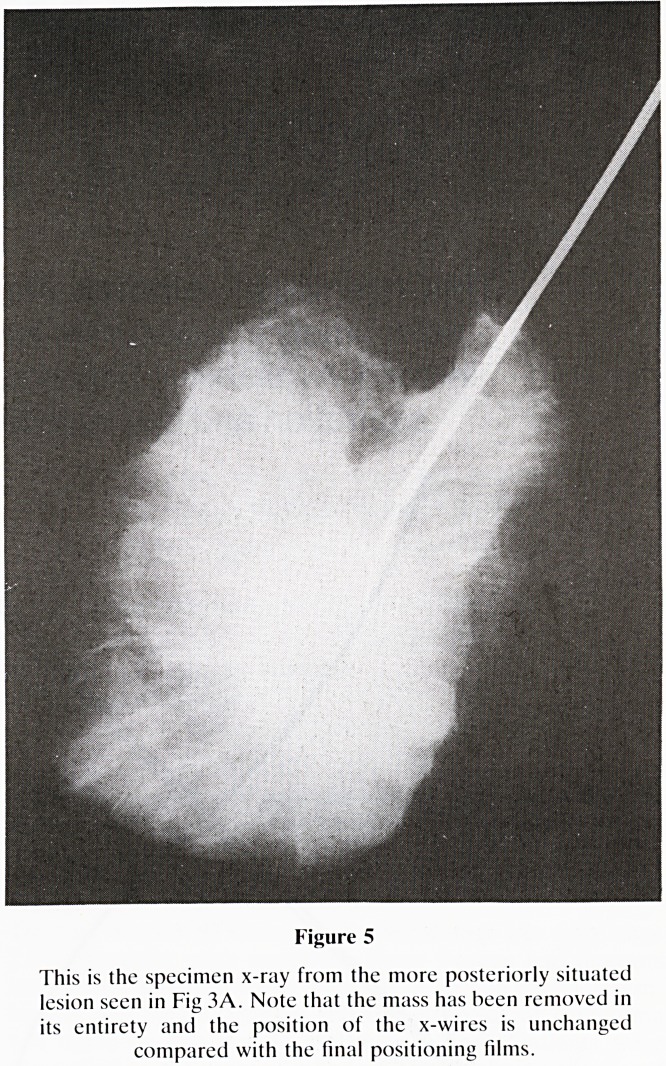# Preoperative Localisation of Impalpable Breast Lesions

**Published:** 1989-05

**Authors:** J. F. Reidy

**Affiliations:** Consultant Radiologist, Guy's Hospital, London


### Preoperative Localisation of Impalpable Breast Lesions

**J. F. Reidy**
Consultant Radiologist, Guy's Hospital, London

It is now well established that mammography is a sensitive technique for the detection of impalpable breast lesions and it is the only screening method commonly accepted to be of any real value. In the aftermath of the Forrest report (1) a limited mammography screening programme has been established in the UK. This will mean that many more women, of whom a majority will be asymptomatic, will present with a suspicious abnormality that the surgeon is unable to palpate even when he is aware of its location on the mammogram. The abnormality will usually be a mass like lesion or a focus of microcalcification.

Though the specificity in a screening population will be fairly high because of the relatively low incidence of carcinoma, the positive predictive value of most reported series has been around 10–30%. (2&3). This will mean that 70–90% of the positives following screening mammography will turn out to be benign and many will need a biopsy. This has been a major criticism to the employment of screening mammography and it is very important that when the breast lesion is biopsied the lesion is precisely localised and that the least amount of normal breast tissue is removed. Attempted removal without localisation often leads to the removal of a large amount of breast tissue and a deformed breast, or not uncommonly, the lesion is missed. A variety of breast localisation techniques have been employed (4).

### NON-INVASIVE TECHNIQUE

This method was used before the development of needle localisation devices. It consisted of taking measurements from mammogram films and transposing them onto the breast. One major problem of this method was the very marked difference that exists in the breast compressed at mammography and the supine breast prior to surgery.

### NEEDLE TECHNIQUES

Here a needle is positioned with its tip in the lesion having being guided by measurements taken from conventional mammography films. Usually the needle would be passed into the breast pointing towards the chest wall. In addition to needle displacement there is the risk of the needle going to deep into the breast and into the chest wall. Some operators have advocated injection of blue dye sometimes combined with contrast media when the needle has been positioned either at the time of localisation or immediately prior to surgery. Though these techniques may still have their advocates (4), most radiologists now will use some form of needle hook wire localisation device.

### HOOK WIRE LOCALISATIONS

Though the majority of breast localisation is performed using mammography in some instances ultrasound localisation may be preferred or centres may prefer it in certain situations. Most commonly patients come down to the x-ray department immediately prior to surgery, but with the development of better hook wire devices that are less likely to move, localisation can now be safely performed on the day prior to surgery.

Even though breast localisation is not a painful procedure, apart from the discomfort of the mammography compression, most women will arrive in the x-ray department in an apprehensive and nervous state. Not only are they worried about the localisation procedure but they are of course concerned that they might have breast cancer. It is very important that these patients are carefully handled and reassured and that the technique is carefully explained to them. Nurses and radiographers can best reassure and explain the procedure before the radiologist starts the localisation procedure and while the initial mammography is being performed. As the patient must remain cooperative and sitting throughout the procedure anything more than minimal sedation is not advisable. It is important to carefully and securely seat the patient on a chair or stool. Doing this and having a nurse in close attendance will make the procedure less disturbing for the patient and also it should mean that the patient is much less likely to move at critical stages of procedure.

### Technique

An advantage of the hook wire device is that the needles are passed into the breast parallel to the chest wall. This should avoid the serious complication that has been described where a localisation device has transgressed into the pleural cavity. (5) The shortest route from the skin entry point to the lesion is preferred, but inferior access is not usually practicable. Mammography with a fenestrated compression plate or a calibrated grid is used with the compression device closest to the lesion. A film is then exposed from which the point on the skin which overlies the lesion can be determined using the holes in the fenestrated compression plate or the grid lines of the calibrated grid (figure 1). It is important to carefully check this entry point and to mark it on the skin. Some do not use local anaesthesia but we feel that it is preferable. Usually with a sharp stiff needle it is not necessary to make a preliminary skin nick but if the needle does not go easily it may be necessary. The needle is passed straight down into the breast at right angles to the compression plate and to a depth which should take its tip to the lesion or better still a little beyond it. The compression plate is then carefully removed taking care not to displace the needle. A further film is taken at a 90° angle to the first view (figure 2). This orthogonal view will show whether the needle tip has passed beyond the lesion or is not in far enough. At this stage minor adjustments may be made without further films but if the positioning is not close further films may be necessary. When it is felt that the needle is at or is fairly close to the lesion a hook wire or some similar device is passed into and out of the needle tip into the breast. At this stage no further adjustments can be made and a final pair of films (figure 3a & b) are taken to show the surgeon where the hook wire is in relation to the lesion seen on the original films. These final films and the original films should be sent to the surgeon detailing where the localisation device is in relation to the lesion. Where possible it is better to demonstrate the films to the surgeon personally. Before the patient leaves the x-ray department the wire protruding from the breast should be lightly strapped down to the breast and covered with a dressing.

### Choice of Hook Wire Localisation Devices

The hook wire most commonly used was first introduced in 1976. Variations have been produced but they essentially consist of a fine 21/22 G needle with a thin wire hooked at its distal end and contained within the needle lumen (figure 4). When the tip of the needle is positioned satisfactorily the

**Figure 1**

Two suspicious stellate mases were found on routine mammography. These were impalpable to the surgeon. On this cranio-caudal view using the fenestrated compression plate, the holes through which the needles need to be passed are worked out using the little metallic marker as a reference point.

**Figure 3a & b**

The x-shaped localisation devices have been passed out through the needles. The final pair of films show the relationship of the devices to the lesions. On the medio-lateral view (3A) the 'x' is a little inferior to the more anteriorly situated lesion. On the cranio-caudal view (3B) the position looks good. The round metallic marker on the wire identifies the point at which the wire passes through the skin.

**Figure 2**

On this medio-lateral view the two piece needle is just at the level of the more posteriorly situated lesion. The sharp trocar has been removed from the more anterior lesion and the cannula is a little beyond it.

hook wire is pushed out into the breast and the needle withdrawn. The shape of the hook then prevents withdrawal. Though they have performed reasonably satisfactorily, problems have occurred with these devices and complications have been described. (5,6,7,8) Breast tissue can sometimes be extremely tough and these thin flexible needles can have problems in penetration. The thin wire used is difficult for the surgeon to palpate and it may be transected during breast biopsy (6). Migration may occur where the whole wire is 'lost' in the breast when the hooked and migrates into the breast with a rachet like movement (7).

A needle with a curved end retractable wire has been advocated. Unlike the hook wire the curved end wire may be withdrawn from the needle and the needle repositioned if necessary (9). This curved wire may have problems passing out into dense and tough breast tissuje and there is a tendency for these devices to be pulled out by a surgeon applying the most gentle traction at surgery.

We have been involved in the development of a new breast localisation device (William Cook, Europe) which we feel offers definite advantages for the surgeon. A larger needle (18 G) is used which may be either a one-piece or a two-piece needle. The localisation device has two forward pointing prongs to prevent any possible forward migration into the breast as well as two backward pointing prongs to stop any withdrawal (figure 4). In addition the wire adjacent to this x-shaped tip is thicker and stiff so that the surgeon can easily palpate it when he gets close to it at surgery. The still more proximal wire is soft and floppy so that the wire may easily be taped to the breast. We have used this new needle on nearly 50 occasions and feel that it offers very definite advantages.

**Figure 4**

Uppermost is the x-localisation wire. Note the thicker and stiffer portion of the wire adjacent to the x-device. The lower device shows a standard hook wire device.

We feel that it can be placed on the day before surgery without the fear that it will move.

### DEVELOPMENTS IN LOCALISATION TECHNIQUES

One of the problems of localisation using conventional mammographic equipment is that after needle placement, compression has to be released and reapplied in the orthogonal plane. This needs to be carefully carried out as there is a risk of displacing the needle. Also the initial needle path and adjustments are all governed by manual adjustments in response to the films.

Now more sophisticated stereo-tactic localisation devices are available which can be added to some mammography equipment. These all work on the same principle of calculating the coordinates of a point using two radiological images taken 30° apart. The instrument will then calculate the precise position and depth at which the needle should be placed so that when the needle of appropriate length is placed through a special needle-holder fitted to the unit localisation to millimetre precision is possible. With the patient still in this position another pair of films can be taken to confirm the final position of the localisation device. Such equipment has an additional role in allowing precise fine needle aspiration biopsy for breast cytology.

### SURGERY AND SPECIMEN RADIOGRAPHY

When the surgeon has removed the lesion, often with the localisation device still in position it is vital that he confirms that the lesion has been removed in its entirety. If the lesion is not obvious on gross surgical inspection, specimen radiography will be necessary (figure 5). When microcalcification was the indication for biopsy the surgeon, pathologist or radiologist should check the specimen x-ray and compare it with the original mammogram. This should confirm that all the foci of microcalcification seen on the film are present in the specimen. Where there is a mass-like lesion specimen radiography may be more difficult as the lesion becomes distorted on removal and on separating it from the background of the breast.

**Figure 5**

This is the specimen x-ray from the more posteriorly situated lesion seen in Fig 3A. Note that the mass has been removed in its entirety and the position of the x-wires is unchanged compared with the final positioning films.

[This is an unusual case with two separate lesions but both of them were infiltrating carcinomas].

### REFERENCES

1. Department of Health and Social Security Breast cancer screening report to the Health Ministers of England, Wales, Scotland and Northern Ireland. London HMSO 1986. (Forrest report).
2. BAKER, L. H. 1982. Breast cancer detection demonstration project: five year summary report. CA **42**, 1–35.
3. KOPANS, D. B. and SWANN C. A. 1988. Observation on mammographic screening and false-positive mammograms AJR **150**, 786–786.
4. FEIG, S. A. Methods and equipment for prebiopsy localisation of nonpalpable breast lesions. In: Moskowitz M. Syllabus, categorical course in breast imaging. Oak Brook IL, Radiological Society of North America 1986: 53–60.
5 BRISTOL, J. B. and JONES P. A. (1981) Transgression of localisation wire into the pleural cavity prior to mammography. *Br. J. Radiol.* **54**, 139–140.
6. HOMER, M. J. 1983 Transection of the localisation hook wire during breast biopsy. *Amer. J. Roentgenol* **141** 929–930.
7. DAVIS, P. S., WECHSLER, R. J., FEIG, S. A., MARCH, D. E. (1988) Migration of breast Biopsy localisation Wire. *Amer J. Roentgenol* **150**, 787–788.
8. HOMER, M. J. and PILE SPELLMAN E. R. (1986) Needle localisation of occult breast lesions with a curved-end retractable wire: Technique & Pitfalls. *Radiology* **161**, 547–548.
9. HOMER, M. J. (1988) Localisation of nonpalpable breast lesions with the curved-end retractable wire: Leaving the needle in vivo. *Amer J. Roentgenol* **151**, 919–920.

## Figures and Tables

**Figure 1 f1:**
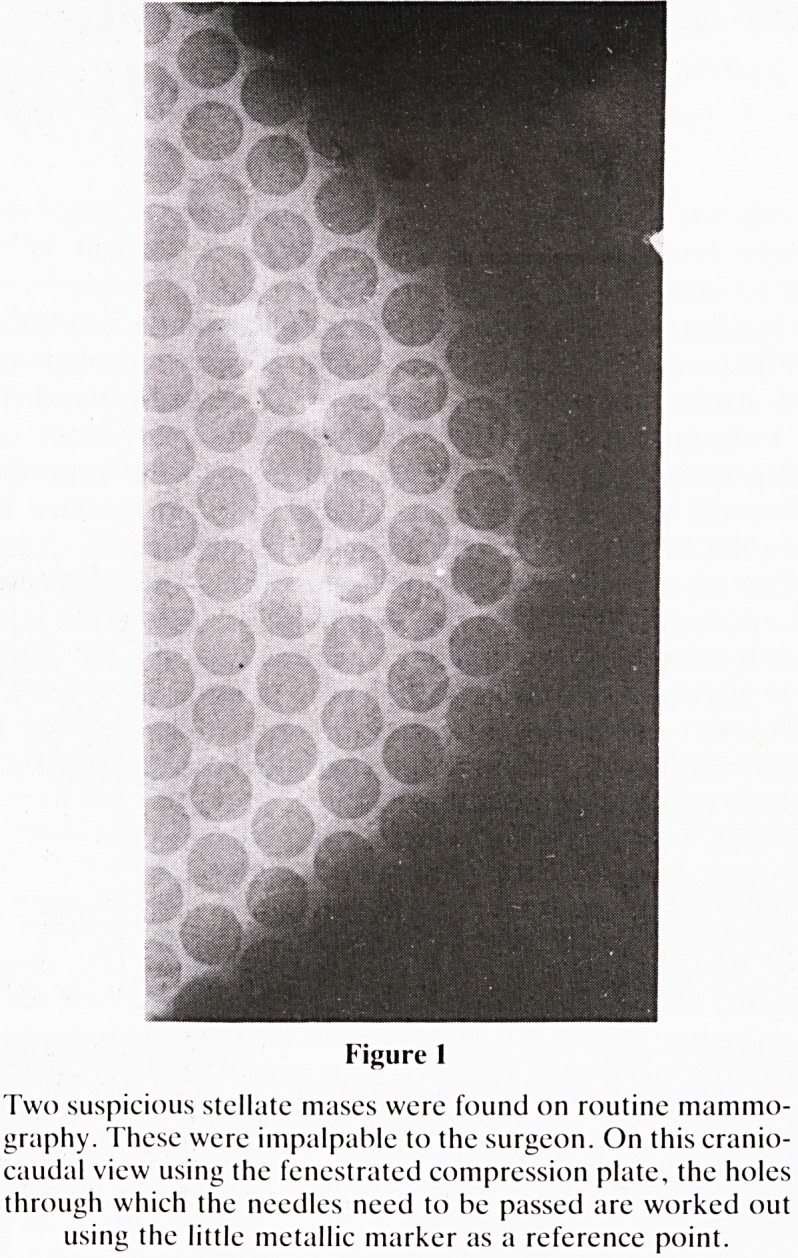


**Figure 2 f2:**
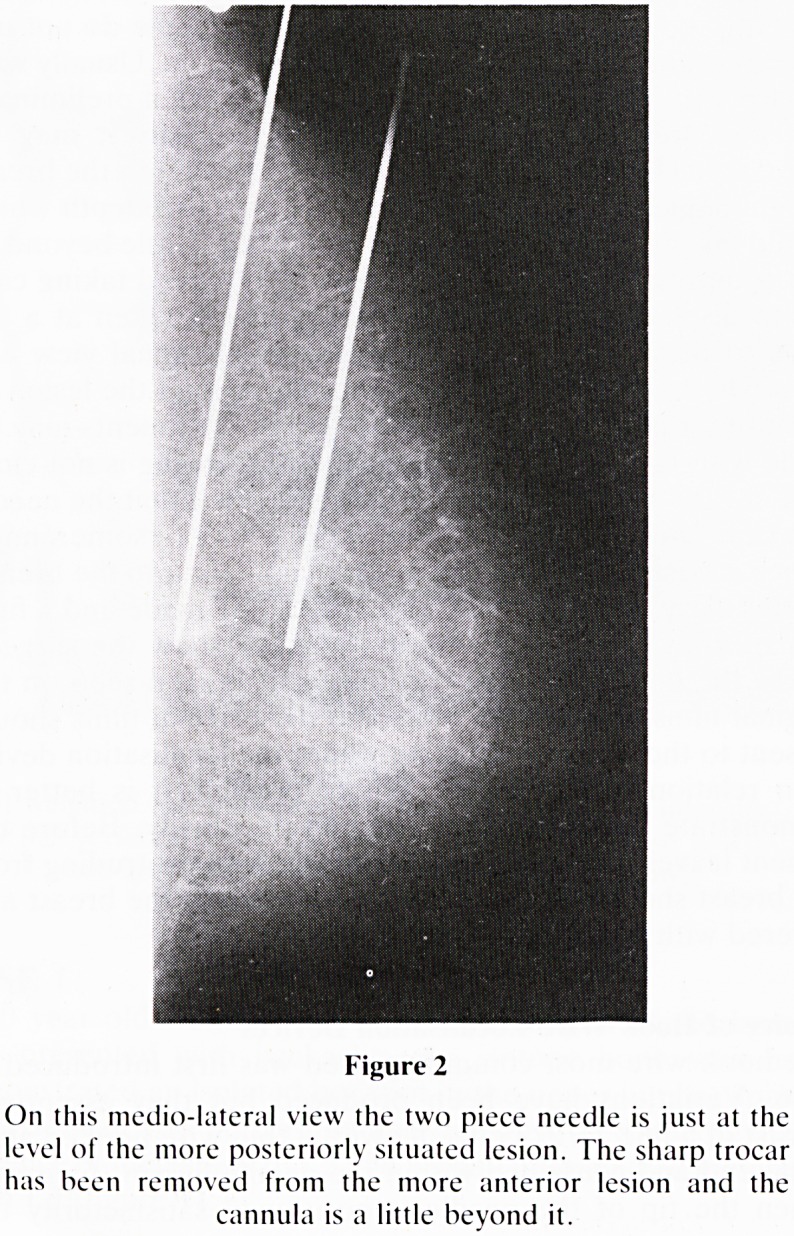


**Figure 3a & b f3:**
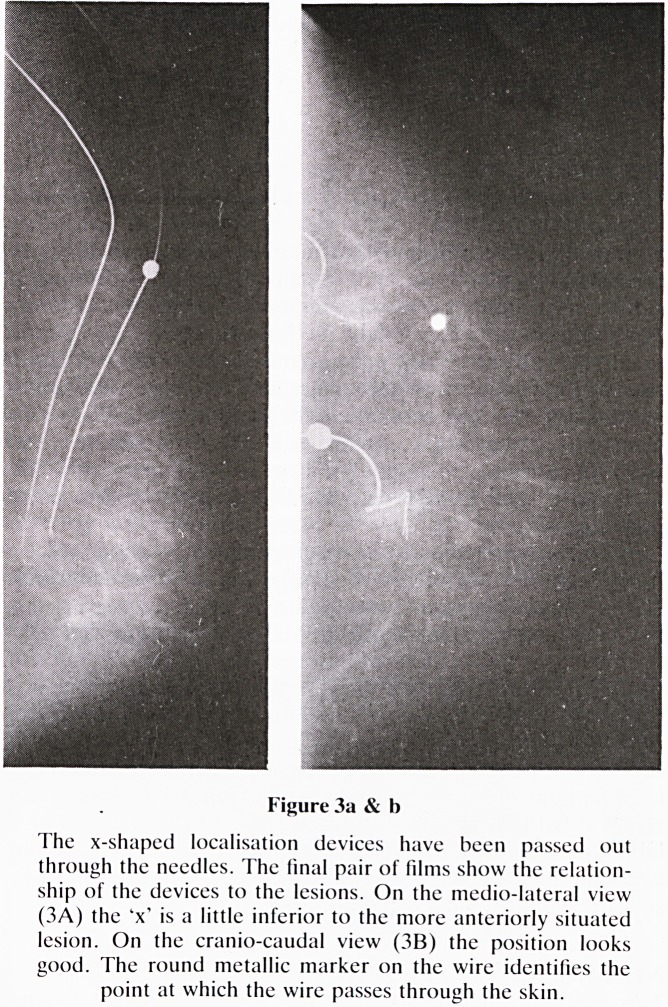


**Figure 4 f4:**
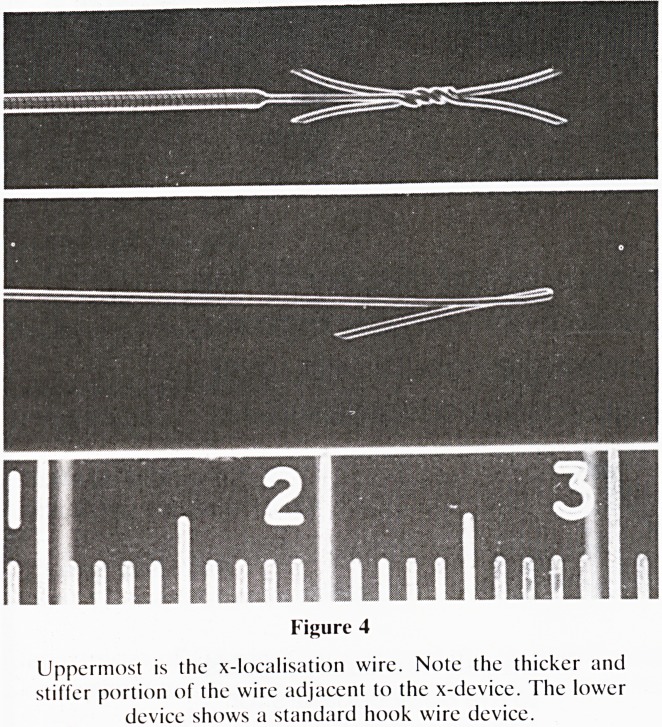


**Figure 5 f5:**